# Oxidative Stress in Treatment-Resistant and Refractory Depression: A Hidden Therapeutic Target?

**DOI:** 10.1007/s12035-026-05715-0

**Published:** 2026-02-05

**Authors:** Zofia Winczewska, Wiesław J. Cubała, Piotr Radziwiłłowicz, Magdalena Górska-Ponikowska

**Affiliations:** 1 Department of Medical Chemistry, Medical University of Gdansk, Gdansk, Poland; 2 Department of Psychiatry, Faculty of Medicine, Medical University of Gdansk, Gdansk, Poland; 3 Department of Biophysics, Institute of Biomaterials and Biomolecular Systems, University of Stuttgart, 70569 Stuttgart, Germany; 4 Euro-Mediterranean Institute of Science and Technology, 90139 Palermo, Italy

**Keywords:** Oxidative stress, Reactive oxygen species, Treatment-resistant depression, Ketamine, Antioxidants

## Abstract

Treatment-resistant depression (TRD) poses a serious challenge to psychopharmacology, as many patients do not achieve remission despite available therapies. The persistence and recurrence of the disease in the absence of response to antidepressant treatment highlight the complex and multifactorial nature of the disease, including the dysregulation of biological processes such as oxidative stress (OS). Although the role of OS in the pathogenesis of depressive disorders has been well documented, a growing body of evidence also points to its potential significance as a biomarker of persistence and symptom severity in TRD. Furthermore, growing evidence suggests that the severity of OS may be a measure of treatment resistance in depressive disorders, shedding light on OS as a potential biomarker of symptom persistence and a therapeutic target in TRD. This article presents the current state of knowledge on the importance of OS as a modifiable risk factor for the severity, chronicity, and persistence of TRD symptoms. Integrating the latest scientific evidence, this review describes the mechanisms linking OS to the development of TRD and discusses fast-acting antidepressants extended by a non-pharmacological nutraceutical approach, which appears to fill a therapeutic gap and increase the chances of recovery for chronically ill individuals. An integrated approach aimed at reducing OS may be a key point of leverage in overcoming treatment resistance in the most severe forms of TRD, thereby contributing to modifying the course of the disease and improving prognosis, which makes this approach the most clinically useful.

## Introduction

Major depressive disorder (MDD) is a pressing public health issue that affects approximately 300 million people worldwide, making it the third leading cause of global health and economic burden [1–3]. It is a heterogeneous disease resulting from many genetic, psychological, environmental, and biological factors, which is consistent with the current biopsychosocial model of depression [4]. The complex and heterogeneous nature of depression makes achieving full treatment efficacy a major challenge, as demonstrated in the STAR*D study, where remission rates declined with successive treatment steps and only 14% of patients achieve remission after three carefully selected and controlled treatment stages, and 15% after four [5]. In addition, patients requiring more treatment steps had higher relapse rates in the later follow-up phase [5]. A particularly difficult subtype of the disease to treat is TRD, which is diagnosed in 30% of patients when a sufficient therapeutic response is not achieved after at least two attempts at antidepressant treatment carried out for an appropriate period and with optimal compliance with medical recommendations [1, 2, 6, 7].

Due to the fact that TRD is not a homogeneous concept and encompasses various phenotypes of depression, the literature also includes definitions such as refractory depression (RD) and difficult-to-treat depression (DTD). RD is a persistent, recurrent subtype of depression characterized by both a lack of response to appropriate pharmacological treatment (characteristic of TRD) and a chronic course persisting in 20–40% of patients for at least 2 years [8, 9]. Patients with RD are characterized by a lower rate of functional remission, higher risk, and shorter time to relapse compared to responders, and a greater number of subsequent therapeutic interventions is associated with a greater clinical burden, including symptom severity and comorbidities [10]. The DTD concept broadens the TRD approach to include somatic, psychosocial, and biological factors, emphasizing not only remission but also improvement in patient functioning and long-term prognosis, which increases its clinical utility [11, 12]. This approach emphasizes the need for optimal symptom control using available therapeutic interventions at various levels, especially in situations where sustained remission is not achievable, and is in line with current recommendations for multidimensional treatment of TRD, including both new, fast-acting antidepressants and effective non-pharmacological somatic therapies [11–13].

Fiorillo et al. also point out that the clinical usefulness of the TRD concept is debatable due to the complexity of the disease and requires a multidimensional interpretation that integrates mechanistic and biological aspects and supports the development of personalized therapeutic strategies [14]. Interestingly, patients who respond to treatment and those with TRD exhibit different symptom spectra. In the group responding to therapy, the so-called affective symptoms predominate, including depressed mood, difficulty concentrating, problems with decision-making, and learning [7]. These are mainly associated with dysfunctions in the serotonergic system, and their resolution under the influence of selective serotonin reuptake inhibitors (SSRIs), which increase central serotonin concentrations, seems to confirm this relationship [7, 15, 16]. However, in patients with TRD, somatic symptoms such as anhedonia, reduced motivation, sleep disturbances, fatigue, lack of energy, and motor slowing seem to predominate. This indicates a link with disorders and dysfunction of the dopaminergic and noradrenergic systems [7, 17, 18]. Although serotonin and norepinephrine reuptake inhibitors (SNRIs), recognized in the treatment of depression, increase the concentration of dopamine and norepinephrine in the central nervous system, they do not guarantee a therapeutic effect in all patients with TRD. This highlights the complexity of depressive disorders, in which disturbed interactions in the monoaminergic system, coexisting with inflammation, one of the mechanisms considered to be significantly associated with drug resistance, play a key role in the etiopathogenesis [7]. The study by Monteleone et al. describes the relationship between the desynchronization of circadian rhythms, which interfere with oxidative, inflammatory, and oxidative pathways, and the development of severe depression, once again pointing to depression as a broad systemic disorder [19]. Furthermore, reduced BDNF concentrations have been associated with severe mood disorders persisting even in a state of euthymia, indicating that neuroplasticity disorders may be an important predisposing factor for the chronicity and persistence of the disease [20, 21]. Patients with TRD also experience so-called residual symptoms of subclinical depression, which often persist in patients with TRD despite meeting the criteria for remission [22, 23]. The persistence of these symptoms indicates incomplete clinical improvement and is considered a predictor of poorer long-term health outcomes, which may ultimately hinder full therapeutic success [24]. Therefore, despite numerous attempts to precisely define TRD, there is still no universally accepted definition that would indicate practical predictive utility allowing for accurate prediction of patient response to subsequent therapeutic strategies [25].

The role of OS in the pathogenesis of depressive disorders is well documented [26, 27], but its potential function as an indicator of symptom severity in TRD is of particular importance. Many studies confirm that excessive production of reactive oxygen species (ROS) and weakening of antioxidant defense mechanisms play a key role in pro-inflammatory signaling, mitochondrial dysfunction, and reduced neurogenesis and neuroplasticity, which appear to be particularly important mechanisms in the development and progression of depression [28–30]. Furthermore, studies have found a positive correlation between the severity of depression and the OS index, indicating that increased OS may reflect the severity and persistence of depressive symptoms [31]. OS may also explain the somatic basis of symptoms characteristic of patients who do not respond to traditional treatment. In the context of the growing problem of TRD, a particular clinical challenge remains the population of patients who do not respond to available therapeutic interventions and who experience exceptionally persistent symptoms. Persistently elevated levels of OS markers may reflect biological “rigidity” of regulatory systems, limiting the body’s ability to restore homeostasis despite pharmacological interventions. Thus, this may lead to the perception of OS as a potential mechanism perpetuating resistance to treatment. Interventions aimed at reducing OS and modulating immunological pathways appear to be particularly interesting.

## Search Strategy

A literature search was conducted in several databases, including PubMed, ScienceDirect, and Google Scholar, to find studies published before October 20, 2025, including only English-language articles. The following terms were used in various combinations: oxidative stress, drug-resistant depression, inflammation, neuroplasticity, major depression, ketamine, and nutraceuticals. This manuscript is a narrative review chosen deliberately because the evidence it contains, including narrative reviews, mechanistic studies, observational studies, and expert recommendations, is too heterogeneous to be synthesized using systematic methods.

## Oxidative Stress as an Indicator of TRD Persistence Severity

OS is a condition resulting from an imbalance between excessive ROS production and insufficient antioxidant activity [32]. ROS include chemically diverse compounds, e.g., hydrogen peroxide (H₂O₂) or superoxide anion radical (O₂•⁻), and the main sources of ROS are the mitochondrial electron transport chain and NADPH oxidases (NOX), which are particularly active in microglia and astrocytes [28, 32–35]. In addition, ROS are produced as a by-product of monoamine oxidase (MAO), an enzyme responsible for the breakdown of monoamine neurotransmitters (serotonin, dopamine, norepinephrine), which play a key role in the mechanisms of depression [26, 36]. Although OS may be a physiological component accompanying the process of neurogenesis in adults [29, 37], high levels of OS are involved in the etiology of many chronic diseases, including neuropsychiatric diseases [38, 39].

ROS, which are highly reactive compounds in excessive concentrations, react with structural elements of the cell, including proteins, nucleic acids, and lipids, causing molecular damage and disrupting cell signaling, thereby disintegrating cell life, including nerve cells [40]. The brain, as a structure with a high content of unsaturated fatty acids, is extremely susceptible to oxidative damage. ROS in the central nervous system (CNS) lead to the peroxidation of unsaturated lipid bonds, forming so-called lipid peroxides, which result in changes in cell membrane functionality, thereby disrupting transmembrane transport and intracellular biochemical homeostasis [41]. Due to the high oxygen turnover in the brain and the high content of transition metals, combined with lower concentrations of antioxidants, the risk of oxidative damage in the CNS is increased compared to peripheral tissues [42, 43].

Impaired balance on the ROS axis and antioxidant defense significantly disrupts neuronal signaling and contributes to the dysregulation of CNS [42]. Furthermore, structural changes in the brain may correlate with the severity of MDD. A study by Klok et al. showed that reduced gray matter volume in the cortex, inferior frontal gyrus, precentral gyrus, angular gyrus, and intraparietal gyrus, as well as specific changes in white matter tracts, particularly in the parietal nucleus, may be characteristic structural features of TRD [43].

### ROS-Induced Inflammation in TRD

**Fig. 1:**
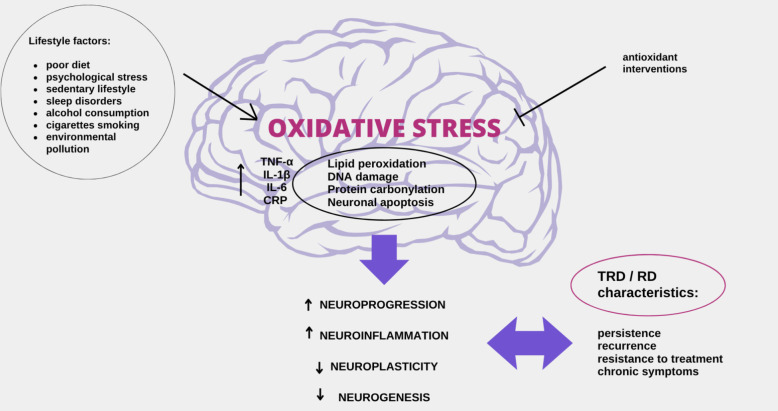
Oxidative stress driven by lifestyle factors influences the persistence of depression through multiple mechanisms

Elevated levels of ROS may also perpetuate neuroinflammation, hyperactivation of the hypothalamic–pituitary–adrenal (HPA) axis, glutamate excitotoxicity, and disruption of brain-derived neurotrophic factor-tropomyosin receptor kinase B (BDNF-TrkB) signaling, which are key neurobiological mechanisms associated with the development of TRD [23, 25]. Neuroinflammation is a mechanism clearly associated with TRD and lack of response to treatment [7, 44–46]. Approximately one-third of patients with depression have low-grade inflammation, which correlates with the percentage of patients with TRD with severe symptoms and prognosis, as well as resistance to treatment [47]. OS remains closely linked to neuroinflammation, and patients with TRD have significantly higher concentrations of inflammatory markers such as tumor necrosis factor α (TNF-α), soluble TNF-α receptor, interleukins (IL) like IL-1β, IL-6, and C-reactive protein (CRP) compared to those who respond to treatment [7, 47–50] (Fig. 1).

Proinflammatory cytokines disrupt the synthesis and release of monoamines in the CNS and enhance their reuptake [7]. Preclinical evidence demonstrates that decreased dopamine promotes induction of the M1 microglial phenotype in the CNS, which is associated with chronic inflammation and increased drug resistance [7, 51–53]. Clinical evidence shows that during severe episodes of MDD, microglial activation is detected in both the prefrontal cortex and the anterior cingulate cortex, with the extent of microglial induction in the anterior cingulate cortex correlating with the clinical severity of depressive symptoms [54, 55]. Importantly, inflammation, by shifting the kynurenine pathway toward neurotoxic derivatives, causes an increase in the ratio between quinolinic acid (QUIN) and kynurenine acid, which promotes susceptibility to the development of depressive episodes, the appearance of symptoms of anhedonia, and a decrease in the effectiveness of antidepressants. Mechanistically, this is associated with neuronal apoptosis but also with increased OS and a reduction in BDNF, which impairs neuroplasticity [7]. Dysfunction of the HPA axis, which may be hyperactive in 50–75% of patients with depression, may also contribute to treatment resistance [30, 45]. Increased ROS modulates HPA axis feedback due to desensitization of glucocorticoid receptors, resulting in its exhaustion and hyperactivity [26, 56]. At the same time, HPA axis hyperactivity increases ROS production, which in turn results in a vicious cycle of OS and HPA axis hyperactivation [48, 57].

OS promotes neuroinflammation by affecting mechanisms related to the modulation of transcription factors such as nuclear factor erythroid 2-related factor 2 (Nrf2), activation of the nuclear factor kappa-light-chain-enhancer of activated B cells (NF-κB) pathway, and stimulation of inflammasomes (NLRP3, NLRP6, NLRC6), which play a significant role in triggering pyroptosis—an inflammatory, programmed form of cell death associated with the pathogenesis of depression [29, 55, 58]. The Nrf2 pathway acts as a cellular defense against OS, which, when exposed to ROS, undergoes translocation from the Keap1 protein complex in the cytoplasm to the cell nucleus, where it binds to the small musculoaponeurotic fibrosarcoma protein (sMaf) and activates the encoding of enzymes that protect against apoptosis and proteins with cytoprotective properties, including IL-10 and BDNF, a signaling protein responsible for neuroplasticity, whose reduced levels are characteristic of depression [29, 59, 60]. Reduced BDNF levels translate into inhibition of Nrf2 nuclear translocation and, consequently, reduced expression of genes involved in encoding antioxidant enzymes, including catalase. This leads to the consolidation of a mechanism in which an increase in ROS levels results in a decrease in BDNF expression and the accompanying depletion of antioxidant reserves [61–63].

In a study based on an animal model, it was observed that the removal of the Nrf2 pathway correlated with reduced levels of monoamines in the prefrontal cortex (serotonin, dopamine) with simultaneously elevated glutamate concentrations, which manifested as depressive symptoms [29]. However, what is particularly interesting is that the addition of rofecoxib treatment reversed depressive behavior, indicating the involvement of the Nrf2 pathway in the induction of inflammatory cascades [29, 64]. This therefore suggests that reduced Nrf2 expression may be a key factor underlying the etiopathogenesis of depression [65]. ROS can initiate ferroptosis, a form of programmed cell death associated with the pathogenesis of depression and indicated as a new potential target for antidepressant therapy [66]. This mechanism remains closely linked to OS, as excessive accumulation of ROS in cell membrane lipids leads to the formation of phospholipid hydroperoxides and damage to the structure of the lipid bilayer [67]. Interestingly, however, the key role of Nrf2 in regulating this process by controlling genes related to glutathione, lipid, and iron metabolism is emphasized [65]. Recent studies also point to the Nrf2 pathway as a promising therapeutic target for counteracting the adverse effects generated by ROS in depression [65], especially since the observed decline in antioxidant defense, including Nrf2-dependent thioredoxin and peroxiredoxin systems, suggests that Nrf2-dependent defense mechanisms against OS are weakened in depression [65].

High levels of ROS also lead to disturbed NF-κB homeostasis [68]. ROS activate the NF-κB pathway through various mechanisms: phosphorylation of inactive IκB protein by IkappaB (IKK) kinase, leading to nuclear translocation of NF-κB [29, 69]; activation of Jun N-terminal kinase (JNK); and extracellular signal-regulated kinase [29, 70]. ROS also influence the activation of inflammasomes, including NLRP3, which are protein complexes responsible for initiating inflammatory processes and promoting pyroptosis [29, 63], a form of programmed lytic cell death, which is associated with depressive disorders and neuroinflammation [71, 72]. In addition, chronic inflammation in people suffering from depressive disorders may reduce the level of negative response protein (NRROS), which, under physiological conditions, is responsible for the degradation of the NOX-2 subunit, part of the NADPH complex responsible for ROS production. Therefore, decreased NRROS levels increase ROS levels, which, through activation of the NF-κB pathway, lead to a vicious cycle of inflammatory cascade [29].

Recent studies also indicated the involvement of the P2X7R receptor in the pathogenesis and progression of depression [73, 74]. As this receptor is involved in the activation of many signaling pathways, it becomes a potential therapeutic target in the treatment of depression, which once again emphasizes the key role of immunoinflammatory pathways in the pathogenesis and severity of depression. OS also leads to astrocyte dysregulation and an imbalance between glutamate and GABA, exacerbating excitotoxicity, which is a characteristic pathophysiological background of TRD [7, 33]. Excess ROS inhibits the expression of the glutamate transporter (GLT-1) in the cell membrane of astrocytes, reducing the ability to remove glutamate from the extracellular space, resulting in neurotoxicity [33, 75]. Conversely, excess glutamate in synapses causes an increase in intracellular calcium concentration, resulting in mitochondrial dysfunction and activation of calcium-dependent enzymes such as death-associated protein kinase 1 (DAPK1) and neuronal nitric oxide synthase (nNOS). Mitochondrial malfunction is also often described as an important factor in the development of depression [76, 77]. Cellular bioenergetic failure translates into the generation of OS, which impairs the viability and functionality of neurons in the CNS and may underlie the structural changes in the brain observed in severe and drug-resistant forms of the disease [26, 76–79]. These processes lead to excessive ROS generation, which, combined with increased protease and lipase activity, promotes OS and cell apoptosis [33, 75]. Furthermore, the N-methyl-D-aspartate (NMDA) receptor activation also translates into reduced synthesis and release of BDNF, lower levels of which are characteristic of patients with TRD compared to those who respond to treatment [7, 80, 81].

**Fig. 2:**
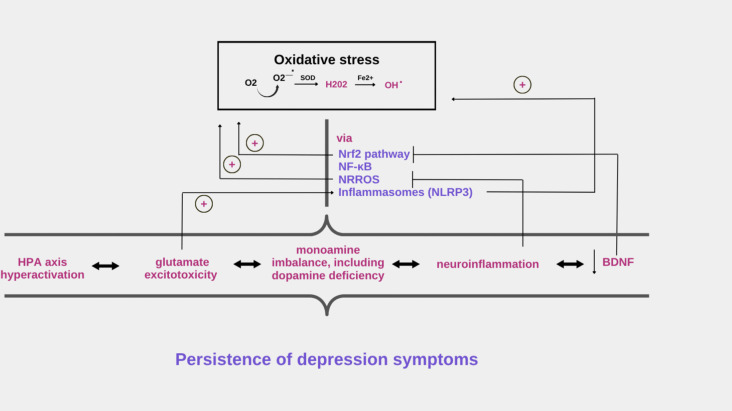
Persistence of depression symptoms via oxidative stress mechanisms. OS by the Nrf2, NF-κB, inflammasome, and NRROS pathways affects the neurobiological basis of depression and the persistence of symptoms characteristic of TRD. Glutamate excitotoxicity also drives OS by enhancing inflammasome activity. Decreased BDNF inhibits Nrf2 pathway activity, leading to increased generation of ROS and the development of OS. Neuroinflammation, by reducing NRROS activity, leads to increased ROS levels, which, through activation of the NF-κB pathway, leads to a vicious cycle of inflammatory cascade, OS, and increased persistence of depressive symptoms

The link between OS mechanisms and the persistence of depressive symptoms is shown in Fig. 2.

The relationship between the activation of inflammatory pathways and the development of depression has been confirmed in numerous observational studies, which have shown a positive correlation between the severity of OS and depressive symptoms, as well as a negative correlation between antioxidant status and the risk of depression [45, 82]. Biomarkers of OS, including 8-isoprostane, malondialdehyde (MDA), and 8-hydroxy-2′-deoxyguanosine (8-OHdG), are elevated in depressive disorders, while the activity of key antioxidant enzymes such as glutathione peroxidase (GPx) and superoxide dismutase (SOD) is reduced [49, 83–86]. Growing evidence suggests that more severe MDD correlates with higher levels of 8-OHdG compared to milder forms of MDD, which may make OS a potential biomarker for the persistence of depressive episodes [87]. Dietary factors further modulate oxidative balance in patients with depression. Lower intake of antioxidants and reduced plasma concentrations of coenzyme Q10, glutathione, vitamins C and E, tocopherols, and polyunsaturated fatty acids have been reported in depressed populations [29, 68, 88]. The results of one meta-analysis showed that vitamin C and vitamin E intake are inversely proportional to depression, highlighting the potential role of diet in supporting antioxidant defenses [89].

The study by Bader et al. used machine learning to suggest that the use of OS biomarkers such as GSH may be an innovative tool to complement diagnostics in the context of predicting the onset of depression and assessing the severity of symptoms [90]. In a recently published study by Winczewska et al., patients with TRD had high levels of hydrogen peroxide, which may be due to abnormal estrogen metabolism inducing OS in this group of patients [55]. Other studies have also confirmed that elevated H2O2 concentrations were characteristic of individuals with recurrent, severe depression [91]. At the same time, reduced SOD concentrations were observed in TRD, which can be explained by a decrease in antioxidant potential associated with high OS [55, 92, 93]. In addition, a positive correlation between total oxidative status and depression severity was also noted, with an inverse relationship in the case of total antioxidant capacity [94]. Since TRD patients typically have high OS levels and reduced responsiveness to conventional pharmacological strategies, this may lead to the perception of OS as a potential mechanism perpetuating treatment resistance. Intervention attempts to overcome OS may therefore improve prognosis and increase the chances of remission.

### Oxidative Stress—Modifiable Factors for Improving the Course and Overall Prognosis

Due to the heterogeneous and complex nature of TRD, effective therapeutic strategies should be based on a multimodal approach, which, in addition to pharmacotherapy, also includes interventions that support its effectiveness, increase the efficacy of treatment, and improve safety in long-term disease management. In the context of OS in TRD, its reduction can potentially be achieved by integrating drugs with antioxidant properties with nutraceutical interventions.

Poor eating habits are considered a modifiable risk factor for depression and its course [95]. The SMILES study showed that adopting a Mediterranean diet with high anti-inflammatory potential improved depressive symptoms and the course of the disease [96]. The benefits of the diet appear to stem from its effect on regulating OS and inflammation, as confirmed by studies showing an inverse relationship between the antioxidant level of the diet and the severity of depression symptoms. [97]. An integrated therapeutic and nutritional approach offers a promising strategy for alleviating symptoms in difficult-to-treat forms of depression, thereby increasing patients’ chances of achieving remission and improving their overall prognosis [98]. Preliminary evidence suggests that lifestyle changes, including micronutrient supplementation and targeted supplementation, may support recovery [99]. There is currently a definition of metabolic psychiatry that refers to an approach focused on identifying and treating metabolic dysfunctions, emphasizing the comorbidity of depression and strictly somatic diseases [100]. Nutraceutical and phytotherapeutic supplementation in depressive disorders based on class A evidence has been presented in guidelines for clinicians by experts from The World Federation of Societies of Biological Psychiatry (WFSBP) and Canadian Network for Mood and Anxiety Treatments (CANMAT) Taskforce [101]. In the context of biomarkers most commonly used to assess OS, glutathione, 8-isoprostane, and 8-hydroxy-2′-deoxyguanosine (8-OHdG) are mentioned. In clinical studies, 8-OHdG in urine or plasma and MDA are often used due to the relative availability of ELISA or HPLC-based tests. Elevated 8-OHdG concentrations are usually interpreted as an indicator of increased oxidative stress, although reference ranges vary and standard cut-off values are not clearly defined. One study suggests that urinary 8-OHdG concentrations exceeding approximately 3.9 ng/mg creatinine (IQR 3–5.5 ng/mg) and values significantly above this may indicate elevated levels of oxidative stress-induced DNA damage. Although SOD activity can also be measured, again methodological heterogeneity and confounding factors limit its routine clinical use, and all markers remain mainly in the research phase in the context of depression [102, 103].

## Rapid-Acting Antidepressants

Ketamine used in the treatment of TRD has a rapid and effective antidepressant effect, which may be at least partly due to its antioxidant component [104]. Ketamine is a rapid-acting antidepressant drug that is a racemic mixture of (*R*)-ketamine and (*S*)-ketamine, which effectively alleviates depressive symptoms in TRD [7, 105]. The mechanism of action of ketamine involves noncompetitive antagonism of NMDA receptors in GABAergic interneurons [30]. By targeting glutamatergic transmission, ketamine in subanesthetic doses exhibits rapid antidepressant effects within 2 h of intravenous administration [106]. Interestingly, however, other compounds that are NMDA receptor antagonists are not effective in reducing depressive symptoms, suggesting that other mechanisms must mediate the antidepressant activity of ketamine. Studies suggest that ketamine has antioxidant activity, as it increases the level of antioxidant enzymes such as SOD and GPx, which are responsible for regulating ROS levels [30, 107]. A study conducted on an animal model showed that a single subanesthetic dose administered to rats under stressful conditions (repeated adrenocorticotropin (ACTH) injections) reduced depressive symptoms through an antioxidant mechanism [30]. Ketamine increased antioxidant defense capacity, with reductions in O₂•⁻, total oxidative stress (TOS), advanced oxidation protein products (AOPP), and MDA, while simultaneously increasing SOD and paraoxonase 1 (PON1) levels. This suggests that the effectiveness of ketamine in depressive disorders may be partly due to its antioxidant activity [30]. Interestingly, and providing contrary evidence, administering ketamine to animals in stress-free conditions had the opposite effect—an increase in MDA levels was observed compared to the control group [30]. The study by Carvalho et al. also confirmed this relationship, suggesting that excessive ROS release, mitochondrial damage, and neurotoxicity may be induced by ketamine under certain conditions, possibly depending on the dose of the drug used [108]. This suggests that the neuroprotective effect of ketamine may be highly dependent on the physiological context, including the severity of the disease and the dose used. Standard therapeutic protocols for TRD typically use an intravenous dose of ketamine of 0.5 mg/kg, which is considered to be within the clinically established therapeutic window. In contrast, preclinical studies have shown that significantly higher doses of ketamine (e.g., 10–20 mg/kg) can simultaneously induce pro-oxidative changes in the central nervous system and anxiety-like behaviors in animal models, suggesting a dose-dependent effect of ketamine, but further well-designed studies are needed [109].

In vitro studies have also demonstrated the neuroprotective effect of ketamine on mouse hippocampal neuronal cells (HT22), significantly reducing oxidative damage induced by glutamate, which mimicked the damage caused by OS [110]. Improvements in mitochondrial function, an elevated ratio of antioxidants to oxidants in cells, and increased expression of the cannabinoid receptor type 1 (CB1) were observed, suggesting that ketamine may reverse the harmful effects of OS on HT22 cells [110]. The results of another study showed that the rapid antidepressant effect of ketamine may result from enhanced neuroplasticity and inhibition of ferroptosis, processes closely related to immunoinflammatory pathways that play a key role in perpetuating the symptoms of depression [111]. A single dose of esketamine in an animal model also reduced lipopolysaccharide (LPS)-induced depressive symptoms by decreasing the expression of proinflammatory cytokines, including TNF-α, IL-1β, and inducible nitric oxide synthase (iNOS), and neuroinflammation in the prefrontal cortex and hippocampus through the activation of Nrf2-dependent anti-inflammatory signaling, which is a promising therapeutic target for counteracting the adverse effects of ROS in depression [65, 112]. In the case of (*R*)-ketamine, preliminary evidence has also emerged of a reduction in behavioral and cellular changes in animal models of neuropsychiatric disorders through anti-inflammatory and antioxidant mechanisms. A reduced increase in pro-inflammatory cytokines and lipid peroxidation in the prefrontal cortex of offspring of mothers exposed to maternal immune activation has been reported [113].

## Nutraceutical Approach in TRD

Basic nutraceutical interventions for MDD have been included in clinical guidelines as a basis for making decisions about supplementation based on evidence-based medicine and are listed in Table 1 [101]. However, phytotherapeutics and nutraceuticals with a documented antioxidant mechanism appear to be particularly interesting.

**Table 1:** Nutraceutical and phytoceutical supplementation as an adjuvant to basic pharmacotherapy in MDD, with the strength of support for specific ingredients rated on a scale: recommended (+ + +), temporarily recommended (+ +), or weakly recommended (+)

Nutraceuticals/phytotherapeutics	Class of evidence and support strength
Omega-3 fatty acids	Class A (+ + +)
Zinc	Class A (+ +)
Probiotics	Class A (+ +)
Vitamin D	Class A (+)
Methylfolate	Class A (+)
S-adenosyl methionine (SAMe)	Class A (+)
St. John’s wort	Class A (+ + +)
Saffron	Class A (+ +)
Lavender	Class A (+ +)
Curcumin	Class A (+ +)

Curcumin is a polyphenol isolated from the rhizome of *Curcuma longa*, which inhibits OS, inflammation, and apoptosis [65, 114]. Turmeric is also a compound included in clinical guidelines as an adjuvant in the treatment of depression, as meta-analyses of studies indicate the effectiveness of curcumin in alleviating the symptoms of depression [115]. The antioxidant effect may mediate the antidepressant effect, as curcumin can stimulate the Nrf2 signaling pathway, among other things, by influencing Nrf2 expression and translocation to the cell nucleus [116], suggesting that curcumin is involved in triggering antioxidant defense.

Growing evidence also points to saffron (*Saffron sativus*) as a raw material that effectively alleviates the symptoms of depression by reducing H_2_O_2_-induced OS. Importantly, current analyses suggest that its antidepressant efficacy may be comparable to that of standard antidepressants, although further well-designed studies are needed to confirm these observations [117, 118].

Omega-3 polyunsaturated fatty acids, especially eicosapentaenoic acid (EPA), known for their anti-inflammatory properties, show potential in the treatment of TRD [119]. Their main mechanisms of action primarily include the reduction of lipid peroxidation and an antioxidant effect resulting from an increase in the antioxidant capacity of cell membranes observed after supplementation. Importantly, these parameters may also be potential biomarkers for predicting response to antidepressant treatment [120].

Supplementation with N-acetylcysteine (NAC), which is an antioxidant, has also been shown to effectively improve treatment outcomes in patients with an insufficient response to standard antidepressant treatment and increased inflammatory activity [121]. NAC, a precursor of glutathione, has been shown to have beneficial effects on, among other things, the modulation of neuroinflammation, the regulation of glutamate activity, and the support of neurogenesis, which appear to be promising targets in TRD.

Studies indicate significantly lower zinc levels in patients with TRD compared to the control group, and lower zinc concentrations may be a marker of TRD and immune response in depression [122]. The results of randomized and observational studies indicated that zinc supplementation reduced the severity of symptoms, but interestingly, a significant improvement in clinical condition was observed only when it was used as monotherapy [123]. A meta-analysis of 52 clinical trials involving 4049 people confirmed that supplementation with antioxidants such as magnesium, zinc, selenium, coenzyme Q10, and crocin has potential as a supplement to traditional antidepressant treatment [124].

Multimodal clinical frameworks integrating biomarkers associated with OS and targeted antioxidant strategies may facilitate better patient stratification and personalized treatment of depression, although their practical application remains limited by the biological heterogeneity of TRD. However, it is recommended to first consider nutritional intervention as one of the early elements of supportive treatment, alongside standard treatment methods. Available evidence suggests that modifying the diet toward a pattern with anti-inflammatory properties and high antioxidant potential may have a beneficial effect on the course of depression [96].

Among the available markers, C-reactive protein (CRP) shows the most consistent associations with inflammation, OS, and symptom severity. Carriers of CRP gene polymorphisms have higher baseline CRP levels in the blood, suggesting that CRP assessment may contribute to treatment stratification and prediction of therapeutic response, as well as aid in decisions about nutraceutical support. However, it is a parameter with insufficient specificity and sensitivity for diagnostic use. Other proposed methods, including the urine pyrrole test, are currently in the preliminary stages and require further validation [125, 126].

## Conclusions and Future Directions

OS is not only a component of the pathogenesis of depression but may also be a key therapeutic target in the most severe, treatment-resistant cases of the disease. Recognizing OS as a modifiable risk factor opens the way to new treatment strategies for TRD. Persistent redox imbalance affects interindividual variability in terms of inflammation activation, neuroplasticity disorders, and neurotransmitter dysfunction, which may partially explain the heterogeneous responses to TRD treatment. Elevated markers of oxidative damage accompanied by reduced antioxidant capacity may identify a biologically distinct subgroup of TRD patients with increased susceptibility to drug resistance. Oxidative stress biomarkers can therefore stratify patients, guide the selection of complementary interventions, or monitor response to treatment, promoting more personalized therapeutic strategies. Beyond the traditional pharmacological treatment regimen, there is an emphasis on the need to integrate lifestyle interventions, including diet, into the management of the symptoms of this disease. Targeted supplementation aimed at overcoming OS can restore oxidative balance, which may aid in the treatment of TRD. Anti-inflammatory models combined with targeted supplementation aimed at overcoming OS may therefore prove to be a complementary strategy that increases the effectiveness of treatment. An integrated approach involving the reduction of OS and the inhibition of inflammatory pathways may contribute to improved therapeutic efficacy and quality of life for patients. Further clinical studies are needed to confirm the efficacy and safety of antioxidant therapies in TRD.

## Data Availability

No datasets were generated or analysed during the current study.
